# tRNA^GlyGCC^-Derived Internal Fragment (i-tRF-GlyGCC) in Ovarian Cancer Treatment Outcome and Progression

**DOI:** 10.3390/cancers14010024

**Published:** 2021-12-22

**Authors:** Konstantina Panoutsopoulou, Tobias Dreyer, Julia Dorn, Eva Obermayr, Sven Mahner, Toon van Gorp, Ioana Braicu, Robert Zeillinger, Viktor Magdolen, Margaritis Avgeris, Andreas Scorilas

**Affiliations:** 1 Department of Biochemistry and Molecular Biology, Faculty of Biology, National and Kapodistrian University of Athens, 15771 Athens, Greece; kpanut@biol.uoa.gr; 2 Clinical Research Unit, Department of Obstetrics and Gynecology, School of Medicine, Technical University of Munich, 81675 Munich, Germany; tobias.dreyer@tum.de (T.D.); julia.dorn@tum.de (J.D.); viktor.magdolen@tum.de (V.M.); 3 Molecular Oncology Group, Department of Obstetrics and Gynecology, Comprehensive Cancer Center-Gynecologic Cancer Unit, Medical University of Vienna, 1090 Vienna, Austria; eva.obermayr@meduniwien.ac.at (E.O.); robert.zeillinger@meduniwien.ac.at (R.Z.); 4 Department of Gynecology, University Medical Center Hamburg-Eppendorf, 20246 Hamburg, Germany; sven.mahner@med.uni-muenchen.de; 5 Department of Obstetrics and Gynecology, Division of Gynecologic Oncology, University Hospital Leuven, Leuven Cancer Institute, 3000 Leuven, Belgium; toon.vangorp@uzleuven.be; 6 Department of Gynecology, Campus Virchow, Charité University Medicine, 13353 Berlin, Germany; ioana@braicu.de; 7 Laboratory of Clinical Biochemistry—Molecular Diagnostics, Second Department of Pediatrics, School of Medicine, National and Kapodistrian University of Athens, “P. & A. Kyriakou” Children’s Hospital, 11527 Athens, Greece

**Keywords:** non-coding RNAs (ncRNAs), transfer RNAs, tRNA-derived small fragments (tsRNAs), tRFs, tRNA fragments, internal tRFs, itRF, molecular diagnostics

## Abstract

**Simple Summary:**

In the precision medicine era, epithelial ovarian cancer (EOC) is characterized by a high death-to-incidence rate and poor 5-year survival. The identification of novel molecular markers is of utmost importance to guide personalized prognosis. The objective of the present study has been to evaluate, for the first time, the prognostic utility of tRNA-derived fragments (tRFs) in ovarian carcinomas. In this context, we have performed in silico analysis and expression profiling, utilizing a TCGA-OV database, GEO datasets and our two institutionally-independent cohorts. The analysis highlighted the internal tRF derived from tRNA^GlyGCC^ (i-tRF-GlyGCC) as a novel molecular predictor of EOC prognosis. More precisely, elevated i-tRF-GlyGCC levels were correlated with an aggressive phenotype of ovarian tumor and linked to adverse survival outcomes and early progression following debulking surgery and platinum-based chemotherapy. Interestingly, i-tRF-GlyGCC integration in multivariate strategies benefits prognostication and achieves superior patient risk-stratification, supporting precision medicine decisions.

**Abstract:**

Epithelial ovarian cancer (EOC) remains a highly-lethal gynecological malignancy, characterized by frequent recurrence, chemotherapy resistance and poor 5-year survival. Identifying novel predictive molecular markers remains an overdue challenge in the disease’s clinical management. Herein, in silico analysis of TCGA-OV highlighted the tRNA-derived internal fragment (i-tRF-GlyGCC) among the most abundant tRFs in ovarian tumors, while target prediction and gene ontology (GO) enrichment analysis predicted its implication in key biological processes. Thereafter, i-tRF-GlyGCC levels were quantified in a screening EOC (*n* = 98) and an institutionally-independent serous ovarian cancer (SOC) validation cohort (*n* = 100, OVCAD multicenter study). Disease progression and patient death were used as clinical endpoints for the survival analysis. Internal validation was performed by bootstrap analysis and the clinical net benefit was estimated by decision curve analysis. The analysis highlighted the significant association of i-tRF-GlyGCC with advanced FIGO stages, suboptimal debulking and most importantly, with early progression and poor overall survival of EOC patients. The OVCAD validation cohort corroborated the unfavorable predictive value of i-tRF-GlyGCC in EOC. Ultimately, evaluation of i-tRF-GlyGCC with the established/clinically used prognostic markers offered superior patient risk-stratification and enhanced clinical benefit in EOC prognosis. In conclusion, i-tRF-GlyGCC assessment could aid towards personalized prognosis and support precision medicine decisions in EOC.

## 1. Introduction

Ovarian cancer (OC) is the most lethal female reproductive system-related malignancy in developed countries, responsible for more than 300,000 incidences and 200,000 deaths worldwide per year [1,2]. Diverse molecular characteristics and clinical behaviors render this cancer a highly heterogeneous disease comprising of several histological subtypes [3]. Epithelial ovarian cancer (EOC) represents the largest subgroup (90%) of ovarian neoplasms and is further subdivided into five major histotypes: high-grade serous (70%), endometrioid (10%), clear cell (10%), low-grade serous (<5%) and mucinous (3%); each of them encompassing their own morphological and molecular features [4,5].

Throughout the last decades, major improvements have been accomplished in patients’ survival in many human malignancies; however, only a modest improvement was observed in EOC, while emerging novel strategies remain suboptimal. In fact, the newly diagnosed EOC patients are treated with the one-size-fits-all approach of cytoreductive surgery and adjuvant platinum-based first-line chemotherapy, while disease prognosis relies mainly on FIGO stage and residual tumor size following debulking surgery [6,7]. Nonetheless, the majority of women will progress after first-line chemotherapy and maintenance therapy, which constitutes the main reason for the poor prognosis of EOC patients [8,9]. To overcome recurrence, the emergence of targeted therapies, such as the FDA-approved bevacizumab (monoclonal antibody against VEGF-A) and olaparib (PARP inhibitor), envisions ameliorating patient survival and quality-of-life [10]. Beyond doubt, the development of a biomarkers-based approach [11] towards the improvement of prognostic accuracy and risk-stratification of EOC patients will certainly support personalized treatment decisions and modern precision medicine.

Although primarily considered random by-products of tRNA degradation and/or biogenesis, recent advances in RNA biology and high-throughput sequencing clearly highlighted that tRNA-derived fragments (tRFs) constitute an abundant and evolutionarily conserved category of small RNAs with a precise sequence, characterized by specific expression patterns and biological roles [12]. tRFs, 14–40 bases in length [13], are produced by the specific cleavage of primary or mature tRNA molecules [14], and are subdivided into several groups according to their generation; tRNA-derived small RNAs (tsRNAs) emerge from the 3′-end of pre-tRNAs by RNase Z cleavage [15], 3′-tRFs are generated by Dicer or Angiogenin cleavage in TψC-loop of mature tRNAs 3′-end, 5′-tRFs derived mainly by Dicer cleavage in the D-loop of mature tRNAs 5′-end and internal tRFs (i-tRFs) arise from cleavage within internal sites of mature tRNAs. Additionally, tRNA-halves, usually detected under stress conditions, arise from angiogenin cleavage within the anticodon loop [16,17].

It is increasingly apparent that tRFs exhibit crucial roles in numerous biological processes, such as mRNA stabilization, miRNA-like post-transcriptional silencing and regulation of protein translation [18]. Interestingly, an ever-growing number of studies have documented the deregulation of tRFs in numerous human malignancies, along with their active involvement in cancer onset and progression [19]. Focusing on OC, a tRNA^Gly^-derived 5′-tRF (tRF-03357) has been reported to enhance cell proliferation, migration and invasion in vitro [20], while tRNA^Gly^-derived i-tRFs have been identified as potential diagnostic tools since they are differentially expressed in serum OC patients compared to healthy controls and are able to predict OC with high specificity and sensitivity [21]. Recently, their diagnostic capability has been further supported, since numerous tRFs were identified to be differentially expressed in high-grade serous OC (HGSOC) vs. normal ovary tissues [22]. Evidently, clinical assessment of tRFs in EOC could provide novel molecular markers that could successfully face the unmet clinical challenges of personalized prognosis and tailored therapeutics.

The aim of the present study was to analyze, for the first time, the prognostic value of tRFs in EOC. In this regard, following in silico analysis of TCGA-OV and GEO deposited tRF datasets, we targeted tRNA^GlyGCC^-derived internal fragments (i-tRF-GlyGCC) for complete clinical evaluation in two institutionally-independent EOC patient cohorts [23,24].

## 2. Materials and Methods

### 2.1. Screening Cohort

The screening cohort of our study consisted of 98 patients with primary EOC. Histological subtypes were represented by 64 high-grade serous, 14 low-grade serous, 9 mucinous, 6 endometrioid, 2 clear cell carcinomas and 3 undifferentiated carcinomas. All samples were acquired from the Department of Obstetrics and Gynecology, School of Medicine, Technical University of Munich, Munich, Germany. Following radical cytoreductive surgery, tissue samples were fresh frozen and stored at −80 °C until analysis. Platinum-based therapy was given in 97 patients, 1 patient received taxane (paclitaxel) monotherapy, while 14 patients received neo-adjuvant therapy. Disease progression and response to chemotherapy were assessed by CT scan and serum CA125. The study complied with the ethical standards of the 1975 Declaration of Helsinki, as revised in 2008 and approved by the Ethics Committee of the Faculty of Medicine, Technical University Munich (491/17). Informed consent was obtained from all individual participants.

### 2.2. Institutionally-Independent Validation Cohort

One hundred patients with primary advanced (FIGO III/IV) serous OC (SOC) enrolled in the multicenter OVCAD cohort constituted the institutionally-independent validation cohort of the present study [25]. Cytoreductive surgery was performed on all patients and, thereafter, the tissue samples obtained were fresh-frozen and stored in liquid nitrogen until analysis. Platinum-based first-line chemotherapy was applied to patients in accordance with consensus recommendations, while neoadjuvant therapy was administrated to 14 patients. RECIST criteria or CA125 variations (GCIG-criteria) were used to assess response to treatment and progression during follow-up [26,27]. The approval of the presents study’s protocol was obtained by the local Ethics Committees of the participating OVCAD partners (EK207/2003, ML2524, HEK190504, EK366, and EK260), while the study was in agreement with ethical standards of the 1975 Declaration of Helsinki, as revised in 2008. Finally, all participating patients had given informed, written acquiescence.

### 2.3. Extraction of Total RNA

Total RNA was isolated using TRI-Reagent (Molecular Research Center, Cincinnati, OH, USA) from 40–150 mg of homogenized tissue following the manufacturer’s instructions and, thereafter, was dissolved in RNA Storage Solution (Ambion, Carlsbad, CA, USA). RNA concentration and purity were determined spectrophotometrically at 260 and 280 nm, assessing absorbance ratios at 260/280 nm and 260/230 nm, while the quality of RNA was evaluated by agarose gel electrophoresis.

### 2.4. RNA Polyadenylation and First-Strand cDNA Synthesis

Prior to reverse transcription, polyadenylation of 1 μg of total RNA at the 3′-end was carried out using 800 μM ATP and 1 U of *E. coli* poly (A) polymerase (New England Biolabs, Inc., Ipswich, MA, USA), in a 10 μL reaction incubated at 37 °C for 60 min. Polymerase inactivation was accomplished at 65 °C for 15 min. Subsequently, first-strand cDNA was synthesized with 50 U MMLV reverse transcriptase (Invitrogen, Carlsbad, CA, USA), 40 U recombinant ribonuclease inhibitor (Invitrogen) and 0.25 μM oligo (dT) adapt-er 5′-GCGAGCACAGAATTAATACGACTCACTATAGGTTTTTTTTTTTTVN-3′ (V = G, A, C and N = G, A, T, C), in a 20 μL reaction at 37 °C for 60 min. Finally, MMLV was inactivated at 70 °C for 15 min.

### 2.5. Quantitative Real-Time PCR (qPCR)

Quantitative real-time PCR (qPCR) assays based on SYBR-Green fluorescence were optimized to quantify i-tRF-GlyGCC levels. The small nucleolar RNA C/D box 48 (SNORD48), also known as RNU48, served as an endogenous reference control for normalization purposes. Specific forward primers were designed for the RNU48: 5′-TGATGATGACCCCAGGTAACTCT-3′ and for the i-tRF-GlyGCC: 5′-GAGGCCCGGGTTCGATTC-3′, according to published RNA sequences. The specific primers were used along with the universal reverse primer: 5′-GCGAGCACAGAATTAATACGAC-3′ for the generation of specific amplicons. The qPCR assays were performed using 150 nM of each qPCR primer and 2 ng of cDNA, as previously described [23,24]. After amplification, melt curve analysis and agarose gel electrophoresis were carried out to distinguish specific amplicons from non-specific PCR products and/or primer dimers.

### 2.6. In Silico Analysis

The TCGA-OV dataset was analyzed, through MINTbase (https://cm.jefferson.edu/MINTbase/ (accessed on 5 April 2021)) [28,29], while tRF levels from GSE94533 GEO dataset in OC were accessed through tsRBase (http://www.tsrbase.org/ (accessed on 18 May 2021)) [30]. tRFTar (http://www.rnanut.net/tRFTar/ (accessed on 10 April 2021)) [31] was used for i-tRF-GlyGCC target prediction and, thereafter, predicted target gene set was functionally annotated through DAVID v6.8 (https://david.ncifcrf.gov/summary.jsp (accessed on 27 April 2021)) [32] for Gene Ontology (GO) enrichment analysis of biological processes, cellular components and molecular functions. GO analysis was visualized utilizing features of the GOplot package in RStudio [33].

### 2.7. Statistical Analysis

The statistical analysis was performed by the IBM SPSS Statistics 20 software (IBM Corp., Armonk, New York, NY, USA). Sapiro–Wilk and Kolmogorov–Smirnov tests were applied for the evaluation of the normal distribution of the data. Thereafter, the non-parametric Mann–Whitney *U* and Kruskal–Wallis tests were used to correlate i-tRF-GlyGCC levels with clinicopathological features in OC.

Survival analysis was performed by Kaplan-Meier curves, using a log-rank test, as well as uni- and multivariate Cox proportional regression analyses. X-tile algorithm was used for the estimation of optimal cut-off values [34]. Multivariate Cox proportional regression analysis included i-tRF-GlyGCC levels, FIGO stage, tumor grade, residual tumor size, response to chemotherapy and age. Patient death and progression were assessed as clinical endpoint events for the overall survival (OS) and progression-free survival (PFS), respectively. Bootstrap Cox proportional regression analysis based on 1000 bootstrap samples was used for internal validation. 

Ultimately, decision curve analysis (DCA), according to Vickers et al. [35], was performed by the STATA 16 software to assess the clinical net benefit of multivariate prediction models on the patients’ survival.

## 3. Results

### 3.1. i-tRF-GlyGCC Target Prediction and GO Analysis—Association with Adverse Clinical Features in EOC

**Figure 1:**
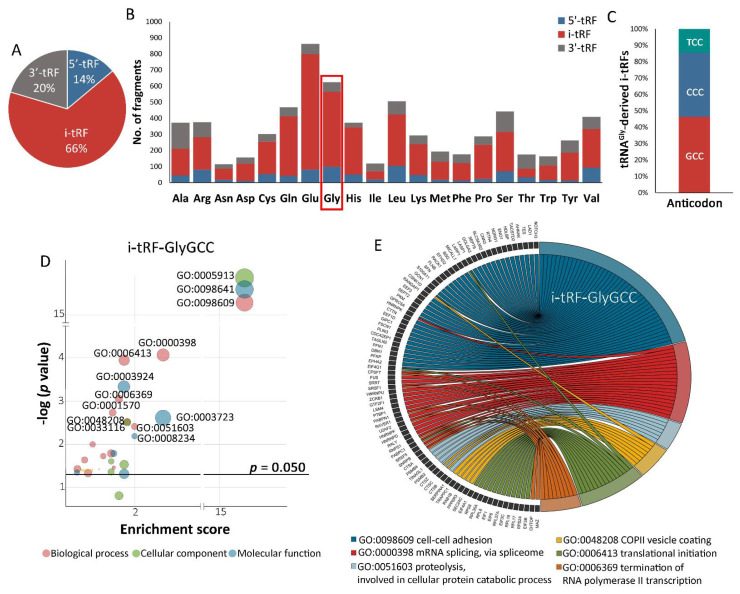
i-tRF-GlyGCC is involved in tumor-promoting pathways in OC. (**A**–**C**) In silico analysis of the TCGA-OV dataset through MINTbase reveals the profiling of tRFs in OC regarding the abundance (**A**), the proportion of fragments derived from distinct tRNAs (**B**) and the frequency of diverse i-tRFs derived from tRNA^Gly^ in OC (**C**). (**D**,**E**) Gene Ontology (GO) enrichment analysis of the predicted i-tRF-GlyGCC targets displayed as a bubble (**D**) and chord (**E**) plot.

TCGA-OV analysis (Figure 1) highlighted that i-tRFs are extensively abundant in ovarian tumors; 66% vs. 14% and 20% of 5′- and 3′-tRFs, respectively (Figure 1A), while tRNA^Gly^-derived fragments revealed to be the second most abundant subgroup, displaying a high proportion of i-tRFs (Figure 1B). Moreover, tRNA^GlyGCC^-derived i-tRFs (GCC anticodon) displayed the highest frequency (~50%) vs. tRNA^GlyCCC^- and tRNA^GlyTCC^-derived i-tRFs (Figure 1C).

Target prediction analysis of i-tRF-GlyGCC by tRFTar enrichment tool identified 574 potential target genes, and GO functional enrichment analysis of the identified target genes by DAVID functional annotation tool resulted in 23 annotation clusters. The distinct terms are illustrated in Figure 1D as a bubble plot. Applying specific inclusion criteria of GO biological processes, *p*-value < 0.05 and enrichment score > 1.3 (in compliance with DAVID recommendations), the implicated genes and the enriched biological processes are plotted as a chord diagram in Figure 1E. Interestingly, the analysis revealed that among the most enriched biological processes are cell-cell adhesion (GO:0098609), mRNA splicing (GO:0000398) and translational initiation (GO:0006413), whose loss of equilibrium represents major hallmarks in ovarian tumorigenesis, disease progression and acquired chemoresistance [36,37,38].

**Figure 2:**
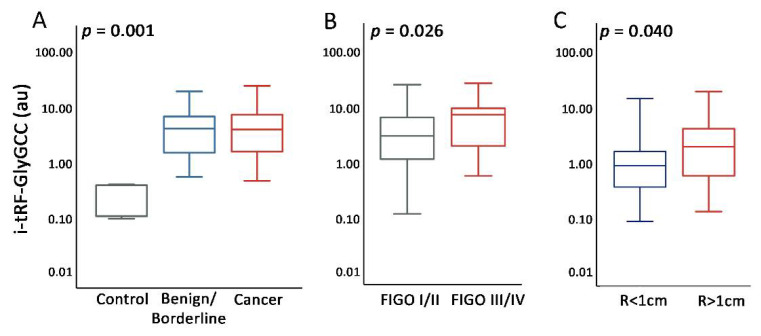
i-tRF-GlyGCC is correlated with unfavorable clinicopathological features in OC. (**A**–**C**) Box-plots presenting the correlation of i-tRF-GlyGCC serum levels in OC patients vs. benign/healthy controls (**A**) and FIGO stages (**B**) in the GSE94533 dataset, as well as of i-tRF-GlyGCC tumor levels with residual tumor size (R) in the screening cohort (**C**). *p*-values were calculated by Kruskal–Wallis (**A**) and Mann–Whitney *U* (**B**,**C**) tests. R: residual tumor size.

To further explore the association of i-tRF-GlyGCC levels with clinicopathological features of EOC, the GSE94533 dataset was utilized [39]. The analysis revealed the significantly elevated serum i-tRF-GlyGCC levels in patients with EOC and benign ovarian lesions compared to normal controls (*p* = 0.001; Figure 2A), as well as the association of i-tRF-GlyGCC elevated serum levels with advanced FIGO stages (*p* = 0.026; Figure 2B). Ultimately, the expression analysis of our screening cohort highlighted the elevated i-tRF-GlyGCC levels in EOC patients with sub-optimal (R > 1 cm) tumor resection (*p* = 0.040; Figure 2C), compared to those with optimal (R < 1 cm) debulking. In this regard, i-tRF-GlyGCC was further targeted to evaluate its clinical value for disease prognosis and treatment outcome, utilizing two institutionally-independent OC cohorts.

### 3.2. Baseline Clinical Data

The screening and validation cohorts consisted of patients with median age 62.0 (range: 25–83) and 61.0 (range: 26–83), respectively, including mostly FIGO III/IV (screening: 85.7%, validation: 100%) and high-grade (screening: 83.4%, validation: 94.0%) tumors. Within a median follow-up time (reverse Kaplan–Meier method) of 93.0 months (95% CI: 80.49–105.51), 98 patients were successfully followed-up in the screening cohort with a median OS of 55.0 (95% CI: 41.52–68.48) and PFS of 22.0 months (95% CI: 16.28–27.72; 2 patients were excluded due to unclear monitoring data), respectively. 

**Figure 3:**
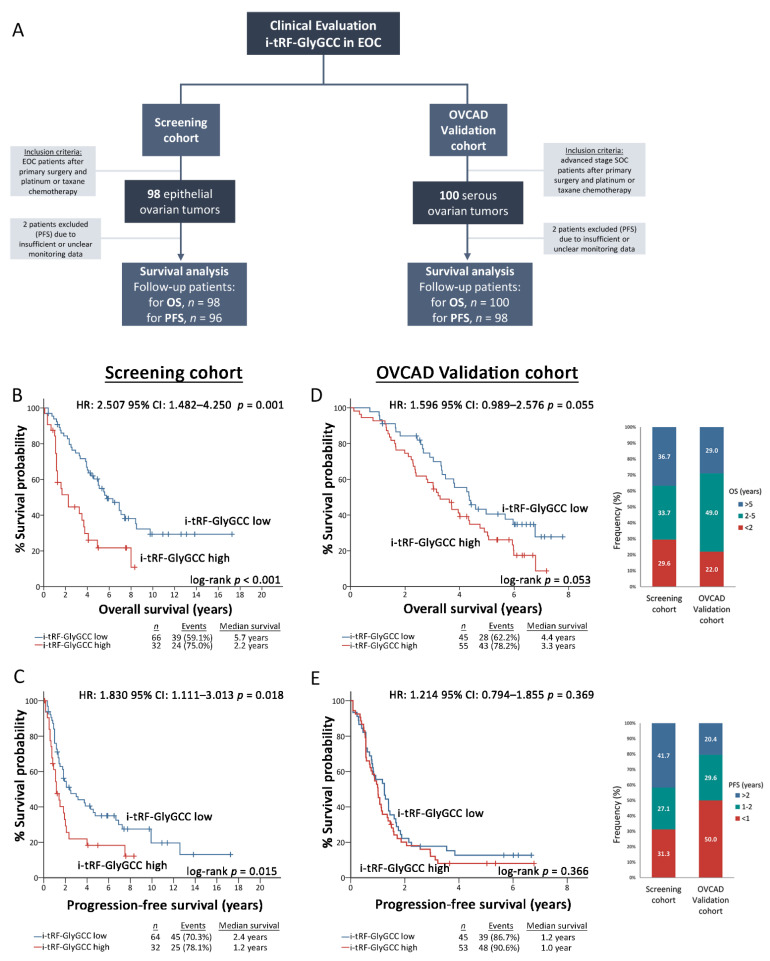
Increased i-tRF-GlyGCC levels predict poor overall survival and early progression of EOC patients. (**A**) REMARK diagram of the study; (**B**–**E**) Kaplan–Meier survival curves plotted based on i-tRF-GlyGCC levels for OS and PFS of the EOC screening cohort (**B**,**C**) and the SOC validation cohort (**D**,**E**). *p*-values were calculated by log-rank test. HR: Hazard Ratio, 95% CI: 95% confidence interval of the estimated HR.

Correspondingly, follow-up was achieved for 100 patients in the OVCAD validation cohort, with a median OS of 45.4 (95% CI: 34.73–56.06) and PFS of 12.24 months (95% CI: 9.36–15.11; 2 patients were excluded due to unclear monitoring data), respectively, during a median follow-up time (reverse Kaplan-Meier method) of 75.56 months (95% CI: 71.76–79.36). Noteworthy, 29.6% of EOC patients in the screening cohort and 22.0% in the OVCAD validation cohort died within the first two years since diagnosis, while 31.3% and 50.0% relapsed within the first year after primary cytoreductive surgery, accordingly. The clinicopathological characteristics of screening and validation cohorts are summarized in Table 1, while the REMARK diagram of the study is presented in Figure 3A), and the complete REMARK checklist is provided in Table S1.

### 3.3. Elevated i-tRF-GlyGCC Levels Are Associated with Unfavorable Prognosis and Treatment Response

Survival analysis was performed using patient death and disease progression as clinical endpoint events for the OS and PFS, respectively. Following the adoption of the optimal cut-off (67th percentile) according to the X-tile algorithm, the screening cohort was divided into the “i-tRF-GlyGCC high” and “i-tRF-GlyGCC low” groups. Interestingly, Kaplan–Meier survival curves revealed a statistically significant correlation of i-tRF-GlyGCC high levels with poor OS (*p* < 0.001; Figure 3B) and shorter PFS intervals (*p* = 0.015; Figure 3C), compared to EOC patients with lower levels. Univariate Cox proportional regression analysis confirmed the significantly higher risk for poor survival (HR: 2.507; 95% CI: 1.482–4.250; *p* = 0.001) and short-term progression (HR: 1.830; 95% CI: 1.111–3.013; *p* = 0.018) of the “i-tRF-GlyGCC high” patients. 

**Figure 4:**
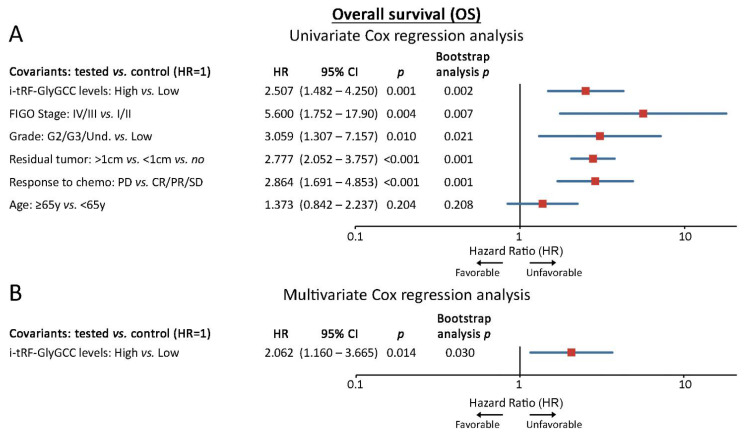
i-tRF-GlyGCC is an independent predictor of overall survival outcome in EOC. Forest plots of the univariate (**A**) and multivariate (**B**) Cox proportional regression analysis for the OS in the screening cohort. Multivariate analysis was adjusted for i-tRF-GlyGCC levels, FIGO stage, tumor grade, residual tumor, response to chemotherapy and age. Bootstrap Cox proportional regression analysis based on 1000 bootstrap samples was used for internal validation. HR: Hazard Ratio, 95% CI: 95% confidence interval of the estimated HR.

**Figure 5:**
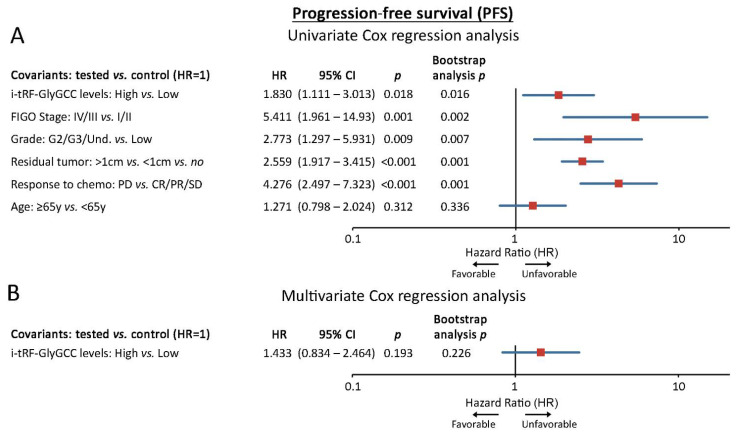
i-tRF-GlyGCC in predicting early-progression in EOC. Forest plots of the univariate (**A**) and multivariate (**B**) Cox proportional regression analysis for the PFS in the screening cohort. Multivariate analysis was adjusted for i-tRF-GlyGCC levels, FIGO stage, tumor grade, residual tumor, response to chemotherapy and age. Bootstrap Cox proportional regression analysis based on 1000 bootstrap samples was used for internal validation. HR: Hazard Ratio, 95% CI: 95% confidence interval of the estimated HR.

Strikingly, multivariate Cox analysis unveiled the ability of i-tRF-GlyGCC upregulation in predicting the worse survival of the patients, independently of FIGO stage, tumor grade, residual tumor after surgery, response-to-chemotherapy and age (HR: 2.062; 95% CI: 1.160–3.665; *p* = 0.014). Internal validation using bootstrap Cox regression models strongly affirmed the powerful and independent unfavorable nature of i-tRF-GlyGCC (Figure 4, Figure 5 and Table S2). To confirm our findings, we have assessed the clinical value of i-tRF-GlyGCC in the OVCAD institutionally-independent cohort. Both Kaplan–Meier curves (*p* = 0.053; Figure 3D) and univariate Cox regression analysis (HR: 1.596; 95% CI: 0.989–2.576; *p* = 0.055) highlighted a substantial trend between the elevated i-tRF-GlyGCC levels and the inferior survival expectancy. No statistically significant association was observed for PFS (Figure 3E).

### 3.4. i-tRF-GlyGCC Ameliorates Patients’ Risk-Stratification and Prognosis

**Figure 6:**
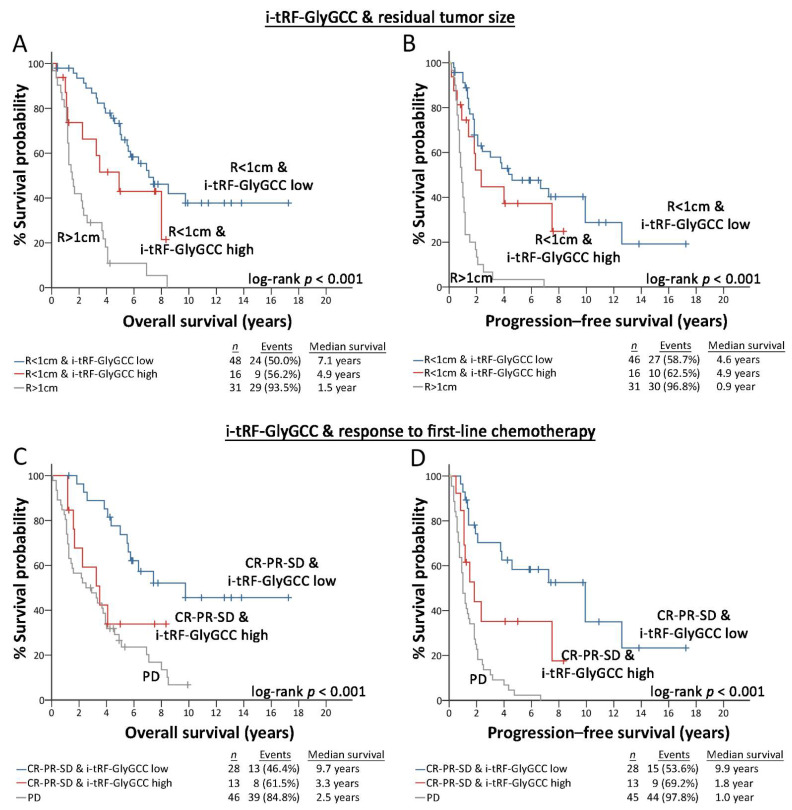
i-tRF-GlyGCC assessment ameliorates patients’ risk-stratification. (**A**–**D**) Kaplan–Meier curves of i-tRF-GlyGCC in compliance with residual tumor size (**A**,**B**) and response to chemotherapy (**C**,**D**) for OS and PFS of the screening EOC cohort. *p*-values were calculated by log-rank test. R: residual tumor size, CR: complete response, PR: partial response, SD: stable disease, PD: progressive disease.

**Figure 7:**
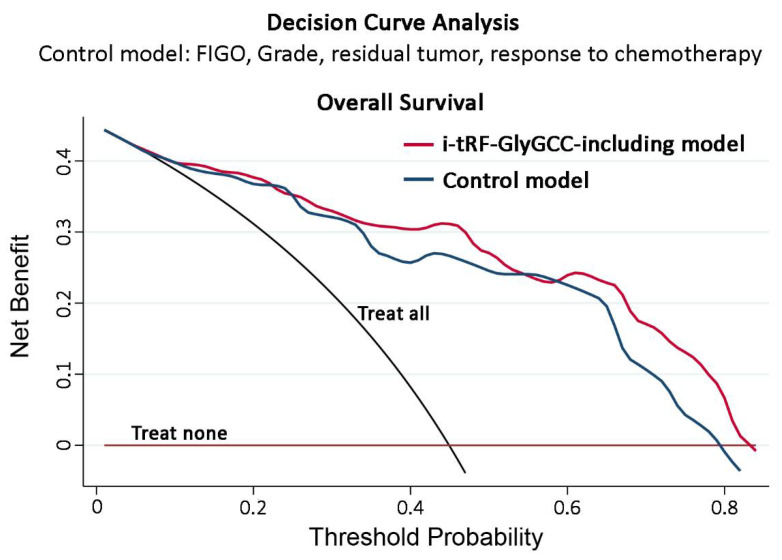
Decision curve analysis highlighted the superior clinical net benefit provided by i-tRF-GlyGCC integration in multivariate strategies. Decision curve analysis of the multivariate prediction models for EOC patients. Net benefit is depicted against various ranges of threshold probabilities for OS.

The disclosed prognostic significance of i-tRF-GlyGCC led us to assess its impact on improving the prognostic value of the established disease clinical markers (Figure 6). The incorporation of i-tRF-GlyGCC levels with residual tumor size and response to first-line chemotherapy unveiled a powerful risk stratification strategy for predicting EOC patient treatment outcomes. Kaplan–Meier analysis revealed that optimally debulked “i-tRF-GlyGCC high” patients are prone to worse survival expectancy (*p* < 0.001; Figure 6A) and disease progression (*p* < 0.001 Figure 6B), compared to optimally debulked patients with lower i-tRF-GlyGCC levels. Moreover, the elevated i-tRF-GlyGCC levels could efficiently define patients with favorable response to first-line platinum-based chemotherapy (CR/PR/SD) at higher risk for poor OS (*p* < 0.001 Figure 6C) and short-term progression (*p* < 0.001 Figure 6D), similar to PD patients, highlighting the superior risk-stratification that integration of i-tRF-GlyGCC can provide in EOC clinical management. Ultimately, DCA was performed to evaluate the clinical benefit of i-tRF-GlyGCC evaluation in EOC prognosis (Figure 7). In this regard, multivariate prognosis prediction model, in-corporating i-tRF-GlyGCC with the clinical disease markers of FIGO stage, tumor grade, residual tumor size and response to chemotherapy resulted in a higher net benefit for patient survival outcome, compared to the model of the clinical markers alone (FIGO stage, tumor grade, residual tumor and response to chemotherapy).

## 4. Discussion

In spite of the major advances in the dynamic field of precision medicine, EOC remains characterized by a high death-to-incidence rate and poor 5-year survival, owing to tumor molecular/histological heterogeneity and chemotherapy resistance [40,41]. The absence of predictive molecular markers that could provide information on the differential clinical benefit in diverse subgroups and allow patient-centric strategies strongly contribute to less personalized treatment decisions [9]. In this context, the quick development of high-throughput technologies, in the last decade, has revealed a novel group of ncRNAs, derived by tRNAs, whose abundance has been documented to vary across tissues and diseases of the same tissue, remaining, however, substantially unchanged within corresponding samples [28]. Unsurprisingly, numerous research groups have identified tRFs as potential prognostic molecular markers in various cancer types [42,43]. Particularly, a high ratio of 5′-tRF-LysCTT/3′-tRF-PheGAA has been correlated with a worse prognosis in prostate cancer [44], while increased 5′-tRF-LysCTT levels were strongly linked to a poor disease outcome in bladder cancer [45]. Additionally, the reduced serum levels of 5′-half-ValCAC were associated with adverse clinicopathological characteristics [46], whereas the elevation of two i-tRFs derived from tRNA^CysGCA^ was correlated to trastuzumab resistance and worse prognosis of breast cancer patients [47]. Ultimately, increased i-tRF-GlyGCC levels have been linked to shorter OS in patients with chronic lymphocytic leukemia [48].

In the present study, the analysis of the TCGA-OV dataset revealed the abundance of i-tRFs and the high proportion of fragments derived from tRNA^GlyGCC^ in ovarian tumors, while GO analysis of i-tRF-GlyGCC predicted targets resulting in enriched key signaling pathways in OC onset and progression, including cell-cell adhesion, mRNA splicing and translational initiation. Additionally, the analysis of the GSE94533 dataset and our screening cohort unraveled the elevation of i-tRF-GlyGCC levels in EOC patients and the association with advanced FIGO stages and suboptimal debulking and prompted us to evaluate its prognostic value in EOC, using our two institutionally-independent cohorts.

Strikingly, i-tRF-GlyGCC emerged as an independent and powerful prognostic indicator in EOC. In particular, the survival analysis of our screening cohort demonstrated that increased i-tRF-GlyGCC levels were significantly correlated with worse survival and higher risk for disease progression in patients that underwent cytoreductive surgery followed by first-line platinum-based chemotherapy. Aiming at reaffirming our findings, we evaluated its clinical significance in the institutionally-independent OVCAD multicenter validation cohort, which highlighted a strong trend between i-tRF-GlyGCC increased levels and the poor survival expectancy of the patients. Moreover, multivariate prognosis models highlighted that i-tRF-GlyGCC evaluation ameliorates the prognostic power of widely-used clinical variables and offers superior risk-stratification of the EOC patients. Remarkably, optimally debulked patients or favorable responders to first-line chemotherapy with elevated i-tRF-GlyGCC levels displayed poor OS and PFS intervals, resembling those of sub-optimal or cPD groups. Ultimately, DCA elucidated the significantly enhanced clinical benefit of the multivariate models combining i-tRF-GlyGCC levels along with disease-established markers in EOC prognostication.

The implication of tRFs in ovarian tumorigenesis has been minimally reported. In particular, a tumor-suppressive role has been documented for 5′-tRF-GluCTC, which inhibits cell proliferation by directly targeting BCAR3 3′-UTR [49]. On the contrary, the oncogenic tRNA^Gly^-derived 5′-tRF (tRF-03357) downregulates HMBOX1 levels and promotes cell proliferation, migration and invasion in HGSOC in vitro [20]. Herein, we have studied, for the first time, the clinical utility of tRFs in EOC, highlighting the unfavorable prognostic value of i-tRF-GlyGCC for EOC patient treatment and survival outcome. Further research of i-tRF-GlyGCC is of utmost importance, as it would uncover its function in ovarian tumor cells and possibly define this tRF as a key effector in EOC tumorigenesis. Identifying i-tRF-GlyGCC deregulation as a driver or downstream event in EOC onset and progression, as well as implementing it in large-scale clinical studies, would undeniably, further unveil its utility as a novel therapeutic target and molecular marker for personalized EOC management and identify the adequate cut-off values for bench-to-bedside approaches. Beyond doubt, the i-tRF-GlyGCC independent predictive value in two institutionally-independent cohorts, with median follow-up time that exceeds five years, as well as the elucidated clinical benefit that can offer in EOC disease prognosis, supports i-tRF-GlyGCC vigorous clinical utility in EOC.

## 5. Conclusions

In conclusion, we have studied, for the first time, the prognostic potential of i-tRF-GlyGCC in two institutionally-independent EOC cohorts. Our data highlighted the unfavorable and independent value of increased i-tRF-GlyGCC levels in predicting short-term progression and poor treatment outcomes of EOC patients. Finally, multivariate prediction models incorporating i-tRF-GlyGCC levels with the established and clinically used disease markers resulted in improved patient risk-stratification, most importantly within the highly heterogeneous groups of optimally debulked patients and chemotherapy responders, and, thus, to superior clinical benefit in EOC prognostication.

## Figures and Tables

**Table 1:** Clinicopathological characteristics of the screening and validation cohorts.

Variable	Screening Cohort No. of Patients (*n* = 98)	OVCAD Validation Cohort No. of Patients (*n* = 100)
**Age**		
<65	57 (58.2%)	62 (62%)
≥65	41 (41.8%)	38 (38%)
**FIGO stage**		
I	11 (11.2%)	-
II	3 (3.1%)	-
III	58 (59.2%)	87 (87%)
IV	26 (26.5%)	13 (13%)
**Tumor**		
T1	14 (14.4%)	2 (2%)
T2	5 (5.2%)	3 (3%)
T3	78 (80.4%)	95 (95%)
Missing data	1	-
**Lymph nodes**		
N0	32 (41.6%)	17 (24%)
N+	45 (58.4%)	54 (76%)
Missing data	21	29
**Metastasis**		
M0	62 (69.7%)	53 (83%)
M1	27 (30.3%)	11 (17%)
Missing data	9	36
**Grade**		
G1	16 (16.5%)	6 (6%)
G2	8 (8.2%)	22 (22%)
G3	69 (71.1%)	71 (72%)
Undifferentiated	4 (4.1%)	-
Missing data	1	1
**Neoadjuvant**		
Yes	14 (14.4%)	14 (14%)
No	83 (85.6%)	86 (86%)
Missing data	1	-
**Ascites**		
none	26 (29.9%)	17 (18%)
<500 mL	19 (21.8%)	31 (33%)
≥500 mL	42 (48.3%)	45 (48%)
Missing data	11	7
**Residual tumor**		
No tumor	45 (47.4%)	68 (68%)
<1 cm	19 (20.0%)	24 (24%)
1–2 cm	17 (17.9%)	1 (1%)
>2 cm	12 (12.6%)	7 (7%)
Inoperable	2 (2.1%)	-
Missing data	3	-
**Response to chemotherapy**		
Progressive disease (PD)	46 (52.9%)	24 (26%)
Complete response (CR)	34 (39.1%)	62 (67%)
Partial response (PR)	7 (8.0%)	4 (4%)
Stable disease (SD)	-	2 (2%)
Missing data	11	8
**Overall survival**		
Follow-up patients	98	100
Alive	35 (35.7%)	29 (29%)
Dead	63 (64.3%)	71 (71%)
**Disease Progression**		
Follow-up patients	96	98
Progression	70 (72.9%)	87 (89%)
Event-free survival	26 (27.1%)	11 (11%)
Missing data	2	2

## Data Availability

RT-qPCR data from the two internal institutionally-independent cohorts are available upon reasonable request. The TCGA-OV and GSE94533 GEO datasets were accessed through MINTbase (https://cm.jefferson.edu/MINTbase/ (accessed on 5 April 2021)) and tsRBase (http://www.tsrbase.org/ (accessed on 18 May 2021)), respectively.

## Supplementary Materials

The following are available online at https://www.mdpi.com/article/10.3390/cancers14010024/s1, Table S1: REMARK checklist, Table S2: Univariate and multivariate Cox regression analysis in the screening cohort.
