# Supplementary material for: tRNAGlyGCC-Derived Internal Fragment (i-tRF-GlyGCC) in Ovarian Cancer Treatment Outcome and Progression
Source: Cancers (Basel). 2021 Dec 22;14(1):24. doi: 10.3390/cancers14010024 (PMC8750938; doi:10.3390/cancers14010024)
Supplement: Supplementary file 1 [file cancers-14-00024-s001.zip › Table S1.pdf]

Table S1. REMARK checklist

ITEM TO BE REPORTED	CHECKLIST	REMARKS	SECTION
**Introduction**			
1. State the marker examined, the study objectives, and any pre-specified hypotheses.	Y	*Marker*: tumor levels of i-tRF-GlyGCC<br>*Study objective*: clinical evaluation of the prognostic value of tRFs in EOC	"Introduction" Section
**Materials and Methods**			
Patients			
2. Describe the characteristics (e.g., disease stage or comorbidities) of the study patients, including their source and inclusion and exclusion criteria.	Y	Screening cohort: n=98 EOC patients; Department of Obstetrics and Gynecology, School of Medicine, Technical University of Munich, Germany (Table 1)<br>Validation cohort: n=100 SOC patients; OVCAD (Table 1)	"Methods" Section:<br>"Screening cohort" & "Institutionally-independent validation cohort"
3. Describe treatments received and how chosen (e.g., randomized or rule-based).	Y	*Administered treatments:* radical cytoreductive surgery & platinum-based first-line chemotherapy	"Methods" Section:<br>"Screening cohort" & "Institutionally-independent validation cohort"
Specimen characteristics			
4. Describe type of biological material used (including control samples) and methods of preservation and storage.	Y	*Biological material:* fresh-frozen tumor specimens<br>*Storage:* -80 ^{o}C until analysis (screening cohort)/liquid nitrogen until analysis (validation cohort)	"Methods" Section:<br>"Screening cohort" & "Institutionally-independent validation cohort"
Assay methods			
5. Specify the assay method used and provide (or reference) a detailed protocol, including specific reagents or kits used, quality control procedures, reproducibility assessments, quantitation methods, and scoring and reporting protocols. Specify whether and how assays were performed blinded to the study endpoint.	Y	- Isolation of total RNA by chloroform-isopropanol extraction<br>- Spectrophotometric evaluation of isolated RNA concentration/purity<br>- Polyadenylation of 3′-end of total RNA<br>- First-strand cDNA synthesis of poly(A) total RNA<br>- SYBR Green-based qPCR specific for i-tRF-GlyGCC quantification<br>Assays performed blinded to the study endpoint.	"Methods" Section:<br>"Extraction of total RNA", "First-strand cDNA synthesis" & "Quantitative real-time PCR"
Study design			
6. State the method of case selection, including whether prospective or retrospective and whether stratification or matching (e.g., by stage of disease or age) was used. Specify the time period from which cases were taken, the end of the follow-up period, and the median follow-up time.	Y	Screening cohort: Retrospective study<br>*median follow-up time:* 93 months<br>Validation cohort: Retrospective study<br>*median follow-up time:* 75.56 months	"Results" Section:<br>"Baseline clinical data"
7. Precisely define all clinical endpoints examined.	Y	*Clinical endpoints:* time-to-death for OS; time-to-progression for PFS	"Methods" Section:<br>"Statistical analysis"
8. List all candidate variables initially examined or considered for inclusion in models.	Y	*Variables examined:* i-tRF-GlyGCC levels, FIGO stage, tumor grade, residual tumor size, response to chemotherapy and age	"Methods" Section:<br>"Statistical analysis"

9. Give rationale for sample size; if the study was designed to detect a specified effect size, give the target power and effect size.	Y	*Sample size rationale:* all available specimens (target power/effect size: ND)	-
Statistical analysis methods			
10. Specify all statistical methods, including details of any variable selection procedures and other model-building issues, how model assumptions were verified, and how missing data were handled.	Y	*Software:* IBM SPSS Statistics 20<br>*Statistical methods:* correlation analysis by non-parametric tests; survival analysis by Kaplan-Meier curves using log-rank test and uni-/multi-variable Cox proportional regression analysis<br>*Clinical net benefit determination:* decision curve analysis (DCA) according to Vickers *et al.* 2006	"Methods" Section:<br>"Statistical analysis"
11. Clarify how marker values were handled in the analyses; if relevant, describe methods used for cutpoint determination.	Y	*Marker values handling:* $2^{-\Delta\Delta CT}$ relative quantification (RQ) method<br>*Cutpoint determination:* X-tile algorithm	"Methods" Section:<br>"Statistical analysis"
**Results**			
Data			
12. Describe the flow of patients through the study, including the number of patients included in each stage of the analysis (a diagram may be helpful) and reasons for dropout. Specifically, both overall and for each subgroup extensively examined report the numbers of patients and the number of events.	Y	Patients' flow through the study is described in REMARK diagram (Fig. 3). Complete REMARK checklist is provided in Table S1.	"Results" Section:<br>"Baseline clinical data"
13. Report distributions of basic demographic characteristics (at least age and sex), standard (disease-specific) prognostic variables, and tumor marker, including numbers of missing values.	Y	The distributions of patients' clinicopathological characteristics are reported in Table 1.	"Results" Section:<br>"Baseline clinical data"
Analysis and presentation			
14. Show the relation of the marker to standard prognostic variables.	Y	i-tRF-GlyGCC is correlated with the presence of the malignancy, advanced FIGO stages and suboptimal debulking (Fig. 2)	"Results" Section:<br>"i-tRF-GlyGCC target prediction and GO analysis – association with adverse clinicopathological features in EOC"
15. Present univariate analyses showing the relation between the marker and outcome, with the estimated effect (e.g., hazard ratio and survival probability). Preferably provide similar analyses for all other variables being analyzed. For the effect of a tumor marker on a time-to-event outcome, a Kaplan–Meier plot is recommended.	Y	Kaplan-Meier curves for OS and PFS in screening and validation cohorts (Fig. 3, 6).<br>Univariate Cox regression analysis for OS and PFS in screening cohort (Fig. 4, 5, and Table S2)<br>Univariate Cox regression analysis for OS and PFS in validation cohort (Fig. 3)	Results" Section:<br>"Elevated i-tRF-GlyGCC levels are associated with unfavorable prognosis and treatment response"

16. For key multivariable analyses, report estimated effects (e.g., hazard ratio) with confidence intervals for the marker and, at least for the final model, all other variables in the model.	Y	Multivariate Cox regression analysis for OS and PFS in screening cohort (Fig. 4, 5, and Table S2).<br>Decision Curve Analysis (DCA) for OS and PFS in screening cohort (Fig. 7).	"Results" Section:<br>"Elevated i-tRF-GlyGCC levels are associated with unfavorable prognosis and treatment response" & "i-tRF-GlyGCC ameliorates patients' risk-stratification and prognosis"
17. Among reported results, provide estimated effects with confidence intervals from an analysis in which the marker and standard prognostic variables are included, regardless of their statistical significance.	Y	Multivariate Cox regression analysis for OS and PFS in screening cohort (Fig. 4, 5, and Table S2).<br>Decision Curve Analysis (DCA) for OS in screening cohort (Fig. 7).	"Results" Section:<br>"Elevated i-tRF-GlyGCC levels are associated with unfavorable prognosis and treatment response" & "i-tRF-GlyGCC ameliorates patients' risk-stratification and prognosis"
18. If done, report results of further investigations, such as checking assumptions, sensitivity analyses, and internal validation.	Y	Internal validation of uni- and multi-variate Cox regression models was performed by Bootstrap analysis based on 1000 bootstrap samples.	"Results" Section:<br>"Elevated i-tRF-GlyGCC levels are associated with unfavorable prognosis and treatment response"
**Discussion**			
19. Interpret the results in the context of the prespecified hypotheses and other relevant studies; include a discussion of limitations of the study.	Y	*Data interpretation*: i-tRF-GlyGCC has been unveiled as an independent molecular predictor in EOC. i-tRF-GlyGCC evaluation ameliorates disease's clinical management.	"Discussion" Section
20. Discuss implications for future research and clinical value.	Y	*Future perspectives:* Unravel the role of i-tRF-GlyGCC in EOC tumorigenesis and incorporate it in multi-institutional large-scale studies to further validate its clinical utility.	"Discussion" Section

Y: Yes; ND: Not Determined.
