# Supplementary material for: tRNAGlyGCC-Derived Internal Fragment (i-tRF-GlyGCC) in Ovarian Cancer Treatment Outcome and Progression
Source: Cancers (Basel). 2021 Dec 22;14(1):24. doi: 10.3390/cancers14010024 (PMC8750938; doi:10.3390/cancers14010024)
Supplement: Supplementary file 1 [file cancers-14-00024-s001.zip › Table S2.pdf]

Table S2. Univariate and Multivariate Cox regression analysis in the screening cohort.

	**Univariate analysis**									
	**Overall survival (OS)**					**Progression-free survival (PFS)**				
**Covariant** tested *vs.* control (HR=1)	**HR**[a]	**95% CI**[b]	***p*-value**[c]	**Bootstrap BCa 95% CI**[d]	**Bootstrap *p*-value**[c]	**HR**[a]	**95% CI**[b]	***p*-value**[c]	**Bootstrap BCa 95% CI**[d]	**Bootstrap *p*-value**[c]
**i-tRF-GlyGCC** High *vs.* Low	2.507	1.482-4.250	0.001	1.502-4.419	0.002	1.830	1.111-3.013	0.018	1.105-3.187	0.016
**FIGO** IV/III *vs.* I/II	5.600	1.752-17.900	0.004	2.010-30.846	0.007	5.411	1.961-14.932	0.001	2.296-26.602	0.002
**Grade** G2/G3/Und *vs.* G1	3.059	1.307-7.157	0.010	1.220-13.053	0.021	2.773	1.297-5.931	0.009	1.239-9.526	0.007
**Residual tumor** >1cm *vs.* <1cm *vs.* no	2.777	2.052-3.757	<0.001	2.121-4.080	0.001	2.559	1.917-3.415	<0.001	1.919-3.951	0.001
**Response to chemotherapy** PD *vs.* CR/PR/SD	2.864	1.691-4.853	<0.001	1.716-5.551	0.001	4.276	2.497-7.323	<0.001	2.649-7.352	0.001
**Age** ≥65y *vs.* <65y	1.373	0.842-2.237	0.204	0.820-2.413	0.208	1.271	0.798-2.024	0.312	0.791-2.075	0.336

	Multivariate analysis[e]									
	Overall survival (OS)					Progression-free survival (PFS)				
**Covariant** tested *vs.* control (HR=1)	**HR[a]**	**95% CI[b]**	***p*-value[c]**	**Bootstrap BCa 95% CI[d]**	**Bootstrap *p*-value[c]**	**HR[a]**	**95% CI[b]**	***p*-value[c]**	**Bootstrap BCa 95% CI[d]**	**Bootstrap *p*-value[c]**
**i-tRF-GlyGCC** High *vs.* Low	2.062	1.160-3.665	0.014	0.978-4.581	0.030	1.433	0.834-2.464	0.193	0.704-2.826	0.226
**FIGO** IV/III *vs.* I/II	1.207	0.343-4.243	0.769	0.263-358254	0.729	2.029	0.588-6.995	0.263	0.268-1199001	0.189
**Grade** G2/G3/Und *vs.* G1	1.949	0.720-5.281	0.189	0.820-9.516	0.181	1.934	0.778-4.810	0.156	0.655-264342	0.154
**Residual tumor** >1cm *vs.* <1cm *vs.* no	2.171	1.530-3.080	<0.001	1.392-4.923	0.002	1.916	1.378-2.665	<0.001	1.219-4.943	0.004
**Response to chemotherapy** PD *vs.* CR/PR/SD	2.197	1.192-4.050	0.012	1.172-5.557	0.013	3.220	1.720-6.027	<0.001	1.637-8.207	0.001
**Age** ≥65y *vs.* <65y	0.830	0.473-1.456	0.516	0.345-1.946	0.593	0.830	0.487-1.413	0.492	0.429-1.505	0.568

[a] Hazard Ratio
[b] 95% confidence interval of the estimated HR
[c] calculated by test for trend. Bootstrap *p*-value is based on 1000 bootstrap samples
[d] Bootstrap bias-corrected and accelerated 95% confidence interval of the estimated HR based on 1000 bootstrap samples
[e] Multivariate analysis adjusted for i-tRF-GlyGCC levels, FIGO stage, tumor grade, residual tumor, response to chemotherapy and age.
